# Unsuppressed viral load and its associated factors among people living with HIV on antiretroviral therapy: a cross-sectional study

**DOI:** 10.1186/s12879-026-13842-1

**Published:** 2026-06-21

**Authors:** Bahejianati Nuerbolati, Yaling Du, Wenying He, Songsong Xie

**Affiliations:** 1 Department of Public Health, The First Affiliated Hospital of Shihezi University, Shihezi, China; 2 Department of Hospital Infection Management, The First Affiliated Hospital of Shihezi University, Shihezi, China; 3 Department of Infectious Disease, The First Affiliated Hospital of Shihezi University, Shihezi, China; 4 National Health Commission(NHC) Key Laboratory of Prevention and Treatment of Central Asia High Incidence Diseases(co-Construction), North Second Road, Shihezi, 832000 China

**Keywords:** Acquired immunodeficiency syndrome, Viral Load unsuppressed, Mediation effects analysis, Risk factors

## Abstract

**Objective:**

This study aimed to assess the prevalence of unsuppressed viral load and its associated factors among people living with HIV (PLWH) receiving antiretroviral therapy (ART) in the Xinjiang Production and Construction Corps (XPCC).

**Methods:**

Clinical data and questionnaires were collected from four divisions of the XPCC via random sampling. Logistic regression and mediation analyses were performed to identify factors associated with unsuppressed viral load.

**Results:**

Among 369 PLWH, 55 (14.91%) had unsuppressed viral load. Associated factors included CD4+ T-cell count ≥ 500 cells/μL, treatment duration ≥ 3 years, alcohol consumption, discrimination, poor ART adherence, and poor self-efficacy (all *p* < 0.05). ART adherence played a full mediating role between self-efficacy, social support and unsuppressed viral load in PLWH, with a mediation effect value (a*b) of β = −0.010 (95% CI: −0.013, −0.006) and β = −0.021 (95% CI: −0.029, −0.014). Greater social support and higher self-efficacy reduce the risk of unsuppressed viral load in PLWH via improved ART adherence. Additionally, the “social support ⇒ medication adherence” pathway exerted a partial chain mediating role between self-efficacy and viral load suppression in PLWH, with a mediation effect value (a*b) of β = −0.012 (95% CI :-0.017, −0.006).Social support can first enhance patients’ self-efficacy, which in turn further improves ART adherence, thereby reducing the risk of unsuppressed viral load.

**Conclusions:**

In the XPCC, the viral suppression rate among PLWH has not yet met the UNAIDS 95–95-95 target. Priority should be given to PLWH with <3 years of treatment and CD4+ T-cell count < 500 cells/μL. HIV-related education should be delivered to individuals, families and the public to enhance social support and self-efficacy, reduce HIV-related stigma, and promote healthy lifestyles (e.g. reduced alcohol consumption). These measures will improve adherence to ART, thereby achieving effective viral load suppression.

## Introduction

Human immunodeficiency virus/acquired immunodeficiency syndrome (HIV/AIDS) remains a major global public health concern that has garnered sustained international attention. As of December 31, 2024, 31 provinces, autonomous regions, and municipalities directly under the Central Government of China had reported a total of 1,355,017 PLWH and 491,437 AIDS-related deaths [1]. The introduction of highly active antiretroviral therapy (HAART) has revolutionized the management of HIV infection and AIDS, effectively transforming it from a fatal condition into a manageable chronic infectious disease. Unlike most other chronic diseases, however, HAART requires lifelong adherence to medication to maintain its therapeutic effects.

UNAIDS has proposed that, by 2030, at least 95% of PLWH who are on treatment achieve viral suppression [2]. The Chinese government has made substantial efforts, and the newly released China Plan for the Prevention and Control of AIDS (2024–2030) [3] reaffirms the commitment to exceed 95% viral suppression rate among treated PLWH by 2030.

The Xinjiang Production and Construction Corps (XPCC), colloquially referred to as the “Xinjiang Corps,” is a provincial-level special administrative unit under China’s separate state planning. As of January 2023, it has jurisdiction over 14 divisions.The XPCC and the Xinjiang Uygur Autonomous Region are two key entities in China’s Xinjiang region, both situated in the country’s northwestern border area, but they differ substantially in administrative level, responsibility scope, and jurisdiction. By the end of 2019, the XPCC had a total population of 3.2484 million. Data indicate that between 1996 and 2021, the XPCC cumulatively identified and reported 1,936 PLWH, including 459 deaths and 1,477 survivors [4]. By the end of 2022, the cumulative number of reported HIV/AIDS cases in the XPCC had risen to 2,130 [5].

According to the *Chinese Guidelines for Diagnosis and Treatment of Human Immunodeficiency Virus Infection/Acquired Immunodeficiency Syndrome (2024 Edition)* [6], after receiving regular antiretroviral therapy for more than 24 weeks, HIV viral load is below the lower limit of detection (<20 copies/mL or 50 copies/mL). This study used whether HIV viral load was below 50 copies/mL as the outcome to determine the current status of unsuppressed viral load among PLWH receiving ART in the XPCC, analyze the associated factors, and provide a basis for improving the effectiveness of HIV antiretroviral therapy in the region.

## Methods

### Study population

Considering factors such as the interviewers’ capabilities, patient cooperation, and the number of PLWH, this study selected four designated HIV antiretroviral therapy hospitals with relatively large numbers of patients within the XPCC jurisdictions to conduct the survey. The survey was carried out from December 2023 to September 2024. After screening for invalid questionnaires, a total of 369 PLWH were finally included as study subjects. Follow-up nurses at the voluntary counseling and testing (VCT) clinics explained the purpose and content of the survey in detail to the PLWH. After the patients signed informed consent forms, the nurses immediately administered the questionnaire survey.

#### Inclusion criteria

1. Meet the diagnostic criteria specified in the Chinese Guidelines for the Diagnosis and Treatment of AIDS (2024 Edition) [6];
2. Be a permanent resident and aged ≥ 18 years;
3. Be receiving ART with a treatment duration of ≥6 months;
4. Voluntarily participate in the survey under the principle of informed consent.

#### Exclusion criteria

1. Cases of being absent from the local area, lost to follow-up, deceased, or in custody at the time of the survey;
2. Patients with severe physical or mental illnesses;
3. Patients who refuse to participate in this survey.

#### Sample size calculation

Based on previous studies, among PLWH, those with detectable viral load accounted for 25% (p = 25%). The sample size was calculated using the formula for cross-sectional studies. ${Z_{\alpha /2}} = 1.96$, *δ * = 0.05. The calculated sample size was 300 cases. Considering a 20% attrition rate, the minimum sample size required for this study was finally calculated to be 360 cases. A total of 369 PLWH were ultimately enrolled in this study.

$$n = {{{{\left( {{z_{\alpha /2}}} \right)}^2} \times P\left( {1 - P} \right)} \over {{\delta ^2}}}$$

### Data collection

Questionnaires were administered during routine follow-up visits. Trained investigators provided standardized instructions, and participants completed the survey only after signing informed consent. Elderly or illiterate participants completed one-on-one interviews; others responded via QR code-based online questionnaires.

Questionnaire content included:

We collected the sociodemographic information and antiretroviral treatment-related data of PLWH using a General and Treatment Information Questionnaire. The Social Support Rating Scale (SSRS) [7]was adopted to investigate the current status of social support among the patients, with a Cronbach’s α coefficient of 0.860, a standardized Cronbach’s α coefficient of 0.873, a Kaiser-Meyer-Olkin (KMO) value of 0.773, and a Bartlett’s test of sphericity with *p* < 0.001. The HIV Treatment Adherence Self-Efficacy Scale (HIV-ASES-V2) [8] was used to assess the self-efficacy of HIV/AIDS patients, which showed a Cronbach’s α coefficient of 0.905, a standardized Cronbach’s α coefficient of 0.925, a KMO value of 0.753, and a Bartlett’s test of sphericity with *p* < 0.001. The Chinese version of the 8-Item Morisky Medication Adherence Scale (MMAS-8) [9] was employed to evaluate the current status of ART adherence in PLWH, with a Cronbach’s α coefficient of 0.830, a standardized Cronbach’s α coefficient of 0.838, a KMO value of 0.716, and a Bartlett’s test of sphericity with *p* < 0.001. Cronbach’s α is an indicator for testing the internal consistency reliability of scales, and with a value between 0.8 and 0.9 indicates excellent scale reliability. The Kaiser-Meyer-Olkin (KMO) test is specifically used to evaluate the construct validity of scales, with a value ranging from 0.7 to 0.79 representing good scale validity. Bartlett’s test is adopted to examine the correlation among scale items.

### Definition of concepts

Alcohol consumption [10]: alcohol consumption was defined as consuming more than 7 drinks per week or ≥ 4 drinks on a single occasion.

Smoking [11]: Persistent or cumulative smoking duration ≥ 6 months, or total cigarette consumption ≥ 100 cigarettes.

Self-efficacy [12]: self-efficacy is defined as an individual’s perceived ability to adhere to HIV treatment regimens, including ART,

### Statistical analysis

Categorical variables were described using frequencies and composition ratios (n/%), while continuous variables were expressed as mean ± standard deviation (*\bar x*±s). For inter-group comparisons, independent samples t-test, analysis of variance (ANOVA), or chi-square test (χ^2^ test) was applied. Binary logistic regression analysis was performed to identify factors associated with viral load unsuppression. Mediating effect analysis was conducted to verify the mediating role of ART adherence in the associations between self-efficacy, social support, and viral load unsuppression.

## Results

A total of 369 PLWH were included in the study, among whom 55 cases (14.91%) had unsuppressed viral load. In terms of demographic characteristics, the subjects were mainly males (301 cases, 81.57%), aged 35–55 years (150 cases, 40.65%), having college education or above (160 cases, 43.36%), having fixed jobs (128 cases, 34.69%), and being married or having partners (175 cases, 43.43%), the main transmission route was heterosexual contact (257 cases, 69.65%), were at disease stage I-II (320 cases, 86.72%), received the ART regimen of NNRTI + 2NRTIs (242 cases, 65.58%), had a CD4+ T-lymphocyte count ≥ 500 cells/μL (170 cases, 46.07%), alcohol use (166 cases, 44.99%), smoking (193 cases, 52.30%), had experienced discrimination (89 cases, 24.12%), and had a history of being subjected to violence (42 cases, 11.38%). (Table 1).

**Table 1:** Univariate analysis of viral load unsuppressed in PLWH

Variables	Total (n = 369)	viral load ＜ 50 copies/mL (n = 314)	viral load ≥ 50 copies/mL(n = 55)	χ^2^	P
**Age (year)**				55.00	<0.01
<35	102 (27.64)	102 (32.48)	0 (0.00)		
35~	150 (40.65)	139 (44.27)	11 (20.00)		
≥55	117 (31.71)	73 (23.25)	44 (80.00)		
**Gender**				0.00	0.95
Female	68 (18.43)	57 (18.15)	11 (20.00)		
Male	301 (81.57)	257 (81.85)	44 (80.00)		
**Educational Level**				34.78	<0.01
Primary School and Below	81 (21.95)	38 (12.10)	43 (78.18)		
Junior High School	38 (10.30)	31 (9.87)	7 (12.73)		
Senior High School and Technical Secondary School	90 (24.39)	86 (27.39)	4 (7.27)		
College Education and Above	160 (43.36)	159 (50.64)	1 (1.82)		
**employment type**				12.05	<0.01
Student	26 (7.05)	26 (8.28)	0 (0.00)		
Farmer/Manual Worker	87 (23.58)	72 (22.93)	15 (27.27)		
With Regular Employment	128 (34.69)	116 (36.94)	12 (21.82)		
Unemployed/Retired/Irregular Employment	128 (34.69)	100 (31.85)	28 (50.91)		
**Marital Status**				18.65	<0.01
Never Married	138 (37.40)	131 (41.72)	7 (12.73)		
Married or in a Cohabiting Relationship	175 (47.43)	140 (44.59)	35 (63.64)		
Divorced/Widowed	56 (15.18)	43 (13.69)	13 (23.64)		
**Transmission Route**				-	<0.01
Heterosexual Sexual Transmission	257 (69.65)	207 (65.92)	50 (90.91)		
Homosexual Sexual Transmission	99 (26.83)	95 (30.25)	4 (7.27)		
Mother-to-Child/Drug Injection/Blood Transfusion Transmission	13 (3.52)	12 (3.82)	1 (1.82)		
**Duration Since HIV Diagnosis (Year)**				4.90	0.03
≤3	140 (37.94)	112 (35.67)	28 (50.91)		
＞3	229 (62.06)	202 (64.33)	27 (49.09)		
**Duration of ART (Years)**				6.47	0.01
≤3	152 (41.19)	122 (38.85)	30 (54.55)		
＞3	217 (58.81)	192 (61.15)	25 (45.45)		
**CD4+ T-Lymphocyte Count(cells/μL)**				68.82	<0.01
<200	46 (12.47)	27 (8.60)	19 (34.55)		
200~	153 (41.46)	127 (40.45)	26 (47.27)		
≥500	170 (46.07)	160 (50.96)	10 (18.18)		
**Disease Stage**				7.20	<0.01
I~II	320 (86.72)	276 (87.90)	44 (80.00)		
III~Ⅳ	49 (13.28)	38 (12.10)	11 (20.00)		
**Adverse Reactions**				5.71	0.02
No	116 (31.44)	107 (34.08)	9 (16.36)		
Yes	253 (68.56)	207 (65.92)	46 (83.64)		
**Treatment Regimen**				0.91	0.82
NNRTI+2NRTIs	242 (65.58)	208 (66.24)	34 (61.82)		
PIs+2NRTIs	40 (10.84)	33 (10.51)	7 (12.73)		
BIC/FTC/TAF	40 (10.84)	36 (11.46)	4 (7.27)		
Others	47 (12.74)	37 (11.78)	10 (18.18)		
**Alcohol Use**				35.15	<0.01
No	203 (55.01)	197 (62.74)	6 (10.91)		
Yes	166 (44.99)	117 (37.26)	49 (89.09)		
**Smoking**				0.52	0.47
No	176 (47.70)	151 (48.09)	25 (45.45)		
Yes	193 (52.30)	163 (51.91)	30 (54.55)		
**Experience of Discrimination**				31.92	<0.01
No	280 (75.88)	273 (86.94)	7 (12.73)		
Yes	89 (24.12)	41 (13.06)	48 (87.27)		
**Experience of Violence**				9.24	<0.01
No	327 (88.62)	285 (90.76)	42 (76.36)		
Yes	42 (11.38)	29 (9.24)	13 (23.64)		
**ART Adherence**	5.25 (3.75, 7.00)	5.75 (4.50, 7.00)	2.50 (2.00, 2.75)	−11.22	<0.01
**Self-Efficacy**	96.00 (85.00, 112.00)	101.00 (91.00, 113.00)	76.00 (74.00, 77.00)	−11.32	<0.01
**Social Support**	48.00 (43.00, 56.00)	50.00 (46.00, 57.00)	37.00 (34.00, 40.50)	−10.00	<0.01

### Univariate analysis of viral load unsuppressed in PLWH

Univariate analysis was conducted with the viral load (VL) results of PLWH designated as the dependent variable. The analysis results demonstrated that several factors were associated with the VL results of PLWH, specifically: age, educational level, occupation type, marital status, transmission route, time of diagnosis, treatment duration, disease stage, CD4+ T-lymphocyte count, adverse drug reactions, alcohol use, experiences of discrimination and violence, ART adherence, self-efficacy, and social support (Table 1).

### Binary logistic regression analysis of unsuppressed viral load in PLWH

Variables that were statistically significant in univariate analysis were included in the multivariate logistic regression analysis. The collinearity diagnostics showed that the variance inflation factors (VIF) for all independent variables ranged from 1.105 to 7.581, all below 10, indicating no severe multicollinearity and confirming the suitability for regression analysis. The Hosmer–Lemeshow test yielded a non-significant result(${\chi ^2} = 2.391,P = 0.566$) indicating good model fit.The results of unconditional binary logistic regression analysis showed that CD4+ T-lymphocyte count ≥ 500 cells/μL (OR = 0.01, 95%CI: 0.00 ~ 0.19, *p* = 0.002), duration of ART ≥ 3 years (OR = 0.05, 95%CI: 0.01 ~ 0.49, *p* = 0.010), alcohol use (OR = 9.25, 95%CI: 2.24 ~ 38.31, P＜0.01), experience of discrimination (OR = 8.50, 95%CI: 1.26 ~ 57.45, *p* = 0.028), ART adherence (OR = 0.15, 95%CI: 0.04 ~ 0.61, *p* = 0.008), and Self-Efficacy (OR = 0.74, 95%CI: 0.60 ~ 0.91, *p* = 0.005) were associated factors for viral load unsuppression in PLWH (Table 2).

**Table 2:** Binary logistic regression analysis of unsuppressed viral load in PLWH

Variables	β	S.E	Z	P	OR (95%CI)
Intercept	11.57	3.82	3.03	0.002	10.64 (5.73 ~ 18.20)
**CD4+ T-Lymphocyte Count(cells/μL)**					
<200	1				
200~	−1.68	1.31	−1.29	0.199	0.19 (0.01 ~ 2.42)
≥500	−4.40	1.40	−3.14	0.002	0.01 (0.00 ~ 0.19)
**Duration of ART (Years)**					
＜3	1				
≥3	−2.97	1.15	−2.58	0.010	0.05 (0.01 ~ 0.49)
**Alcohol Consumption**					
No	1				
Yes	2.22	0.72	3.07	<0.01	9.25 (2.24 ~ 38.31)
**Experience of discrimination**					
No	1				
Yes	2.14	0.97	2.20	0.028	8.50 (1.26 ~ 57.45)
**ART Adherence**	−1.88	0.71	−2.65	0.008	0.15 (0.04 ~ 0.61)
**Self-Efficacy**	−0.30	0.11	−2.81	0.005	0.74 (0.60 ~ 0.91)

### The direct, indirect, and total effects among the relevant factors

In this study, mediation effects were analyzed using the bias-corrected non-parametric percentile bootstrap method with 2,000 resamples. The 95% CI for the path coefficients were examined, and mediation effects were considered statistically significant if the 95% confidence interval did not contain 0. The results showed that the total effect of social support on unsuppressed viral load was −0.055 (SE = 0.020, 95% CI [−0.094, −0.016]). The direct effect of social support on unsuppressed viral load was non-significant (β = −0.004, 95% CI: −0.011, 0.003), whereas the direct effects of self-efficacy (β = −0.004, 95% CI: −0.008, −0.002) and ART adherence (β = −0.088, 95% CI: −0.123, −0.053) on unsuppressed viral load were significant. Regarding indirect effects, ART adherence mediated the relationship between social support and unsuppressed viral load (β = −0.021, 95% CI: −0.029, −0.014); and between self-efficacy and unsuppressed viral load (β = −0.010, 95% CI: −0.013, −0.006); self-efficacy and ART adherence played a chain mediating role in the relationship between social support and unsuppressed viral load (β = −0.012, 95% CI [−0.017, −0.006]); and self-efficacy mediated the relationship between social support and unsuppressed viral load (β = −0.018, 95% CI [−0.027, −0.011])(Table 3).

**Table 3:** Results of Mediation Effect Analysis

	Path	Effect	S.E.	Bootstrap 95%CI
**Direct Effect**	social support→unsuppressed viral load	−0.004	0.004	(−0.011,0.003)
	self-effiency→unsuppressed viral load	−0.004	0.002	(−0.008,-0.002)
	ART Adherence→unsuppressed viral load	−0.088	0.074	(−0.123,-0.053)
**Indirect Effect**	social support→ART Adherence→unsuppressed viral load	−0.021	0.004	(−0.029,-0.014)
	self-efficacy→ART Adherence→unsuppressed viral load	−0.010	0.002	(−0.013,-0.006)
	social support→self-efficacy→ART Adherence →unsuppressed viral load	−0.012	0.003	(−0.017,-0.006)
	social support→self-efficacy→unsuppressed viral load	−0.018	0.004	(−0.027,-0.011)
**Total Effect**	social support→unsuppressed viral load	−0.055	0.020	(−0.094,-0.016)

## Discussion

This study is the first to investigate the current status of unsuppressed viral load among PLWH receiving ART in the XPCC, and to analyze the associated factors of viral load unsuppressed.

The newly released Chinese guidelines for diagnosis and treatment of human immunodeficiency virus infection/acquired immunodeficiency syndrome (2024 edition) [6] states that the assessment of ART efficacy primarily focuses on virological, immunological, and clinical indicators, among which virological improvement serves as the most critical evaluation criterion. It also emphasizes that following the initiation of ART, follow-up evaluations of virological, immunological, and clinical status should be performed every 3–6 months to assess treatment outcomes and guide adjustments to ART regimens. Existing studies [13, 14] have demonstrated that viral load is the optimal predictor of long-term clinical outcomes.

Among the 369 participants, 314 (85.09%) achieved viral suppression, while 55 (14.91%) had unsuppressed viral load. This rate was slightly lower than the 20.80% reported by Qian Y et al. [15]. When compared with other cities in China, it was lower than the rate in Luzhou City, Sichuan Province (25%) [16], in the Dali region of Yunnan Province (15.03%) [17], and the national estimated unsuppressed viral load rate of 17.1% reported by Zhao et al. [18].

This study indicates that a higher CD4^+^ T‑lymphocyte count (≥500 cells/μL) is associated with achieving viral load suppression. Perez-Molina JA et al. [19] demonstrated that baseline CD4^+^ T lymphocyte counts < 200 cells/μL were associated with poor ART outcomes among treatment-naive patients, and this association remained consistent at 48 and 96 weeks after ART initiation. Geoffrey Fatti et al. [20] found that people living with HIV initiating ART with baseline CD4^+^ T lymphocyte counts ≥ 500 cells/μL achieved superior virological outcomes compared with those having counts of 200–499 cells/μL and those with counts below 200 cells/μL.

The results showed that alcohol consumption was associated with unsuppressed viral load, similar to Mannen et al. [21]. The proportion of alcohol consumption among PLWH ranges between 8 and 42% [22]. For PLWH, alcohol consumption not only increases the risk of HIV transmission (e.g., unprotected sex) [22] but also exerts a negative impact on the effectiveness of ART. These adverse effects include reduced ART adherence, failure of immune function recovery, unsuppressed viral load, and even elevated risks of treatment failure and mortality [23]. Additionally, studies by Wynn et al. [24] and Cachay et al. [25] have indicated that alcohol consumption leads to higher viral load by reducing adherence to ART.

This study found that experiencing discrimination is associated with unsuppressed viral load among PLWH, similar to Yigit et al. [26] research. Previous studies have shown that HIV-related stigma is a key barrier to the achievement of the 90–90-90 targets [27]. Specifically, HIV-related discrimination is associated with a reduced willingness to undergo HIV testing among populations engaging in high-risk sexual behaviors, poor linkage between PLWH and healthcare providers, as well as low adherence to treatment and suboptimal viral load suppression among patients. Additionally, the findings of Oryokot et al. [28] and Mukuku et al. [29] have shown that internalized family stigma, external stigma, and discriminatory behaviors are significantly associated with unsuppressed viral load in PLWH.

This study found that higher self-efficacy was associated with better viral load suppression, similar to Umar et al. [30]. Self-efficacy is defined as an individual’s perceived ability to adhere to HIV treatment regimens, including ART, and this ability is associated with better treatment adherence [12]. The prospective cohort observational study conducted by Spence et al. [31] on women living with HIV showed that patients with higher HIV treatment self-efficacy were more likely to achieve viral suppression. The findings of Crockett et al. [32] revealed that depressive symptoms in PLWH indirectly reduce their adherence to ART by diminishing self-efficacy, thereby leading to suboptimal viral suppression. Conversely, the study by Yigit et al. [33] demonstrated that the alleviation of internalized stigma could indirectly improve both ART adherence and viral load suppression in PLWH through enhanced self-efficacy.

This study demonstrated that higher ART adherence is associated with the achievement of viral load suppression among PLWH, similar to Mukuku et al. [34]. ART adherence is defined as strict compliance with the treatment regimen prescribed by healthcare providers, including taking the correct dosage of medication at the right time and in the proper manner [35]. PLWH often exhibit non-adherence to treatment due to reasons such as “forgetting to take medication,” “failing to carry drugs when going out,” “being occupied with other matters,” “fearing disclosure of their condition when outside,” or “choosing to discontinue treatment after improvements in immune function” [36, 37], Such non-adherent behaviors are associated with viral load rebound [34]. A follow-up study conducted by Tchakoute et al. [38] on PLWH in California, USA, demonstrated that ART adherence is significantly associated with both virological and immunological outcomes: every 10% increase in adherence was associated with a 37% higher probability of viral suppression, as well as an average increase of 8.54 CD4 cells/μL within the subsequent six months. Additionally, A study by Glass et al. [39] showed that self-reported missed doses of ART (two or more times) were associated with virological treatment failure and all-cause mortality. In contrast, research by Norcini-Pala et al. [40] demonstrated that ART adherence mediates the relationship between stigma stemming from factors such as poverty, gender, and race and unsuppressed viral load. Conversely, robust social support, high confidence in treatment, fewer ART-related side effects, and adequate nutrition can help patients achieve good treatment adherence, and consistent medication intake is conducive to achieving viral suppression [41].

## Strengths

This study possesses the following strengths. First, it represents the first systematic investigation into the prevalence and associated factors of unsuppressed viral load among people living with HIV (PLWH) receiving antiretroviral therapy (ART) in the Xinjiang Production and Construction Corps (XPCC), providing local evidence for HIV prevention and control in border regions. Second, this study adopts a comprehensive set of variables, encompassing not only demographic and clinical indicators but also behavioral and psychosocial factors, to analyze viral load suppression status among PLWH through a relatively complete framework of factors. Finally, guided by a sound research framework and employing rigorous statistical analytical methods, this study identified the factors associated with unsuppressed viral load among PLWH in the XPCC. The findings can provide a reliable basis for the formulation of subsequent HIV prevention and control strategies, as well as a reference for achieving optimal viral load suppression in other regions.

## Limitations

This study has several limitations. First, as a cross-sectional study, causal inference is limited; future cohort studies may be conducted to clarify the causal relationships among variables. Second, the questionnaire relied on patient self-report, which may introduce reporting bias and recall bias. Third, To ensure data quality and reliable factor analysis, four hospitals with a high volume of PLWH were selected. Accordingly, the observed unsuppressed viral load rate may overestimate the true prevalence in the entire region. Fourth, the effectiveness of antiretroviral therapy is influenced by multiple factors, and unmeasured variables may have affected the results of this study.

## Conclusions

In summary, among 369 PLWH in the XPCC region, 55 cases (14.91%) had unsuppressed viral load, indicating that the current status of unsuppressed viral load still requires improvement. The study identified that CD4+T lymphocyte count ≥ 500 cells/μL, treatment duration ≥ 3 years, history of alcohol consumption, experience of discrimination, ART adherence, and self-efficacy are factors associated with unsuppressed viral load in PLWH. ART adherence is a key factor and can serve as a mediating factor that mediates the effects of social support, self-efficacy, and other factors on viral load.To facilitate the achievement of viral load suppression, targeted measures should include: strengthening precise clinical intervention for key patient groups, enhancing patients’ ART adherence and self-efficacy through diverse approaches, and improving the social support system that combines anti-discrimination publicity with multi-dimensional support.

## Data Availability

The information of PLWH shall be strictly kept confidential.We will require all interested parties to provide the data request in writing. It shall require the approval of both the hospital and the local Center for Disease Control and Prevention (CDC). Once agreed to, data transfer agreements will be developed. All data transfers must comply with all applicable China regulatory requirements. All data usage must comply with all applicable China`s regulatory requirements.

## Abbreviations

PLWH: People living with HIV
XPCC: Xinjiang Production and Construction Corps
HIV/AIDS: Human immunodeficiency virus/acquired immunodeficiency syndrome
HAART: Highly active antiretroviral therapy
ART: Antiretroviral therapy
UNAIDS: Joint United Nations Program on HIV/AIDS
VCT: Voluntary Counseling and Testing
VL: Viral load
NNRTI: Non-Nucleoside Reverse Transcriptase Inhibitor
NRTIs: Nucleoside Reverse Transcriptase Inhibitors
PIs: Protease Inhibitors
